# Patients’ Journey and Burden of Disease Among Patients with Axial Spondyloarthritis in Greece: A Cross-Sectional Online Survey

**DOI:** 10.31138/mjr.240725.ehr

**Published:** 2026-01-08

**Authors:** Katerina Lioliou, Dimitra I. Lampropoulou, Garyfallia Stefanou, George Gounelas, Katerina Koutsogianni, Vasileios Kountouris, Michael Feretos, Georgia Kourlaba

**Affiliations:** 1 ECONCARE LP, Athens, Greece,; 2 PanHellenic Federation Reumazin, Thessaloniki, Greece,; 3 UCB Pharma, Athens, Greece,; 4 Department of Nursing, University of Peloponnese, Tripoli, Greece

**Keywords:** axial spondyloarthritis, surveys and questionnaires, patient journey, burden, diagnostic delays, quality of life

## Abstract

**Objective/Aim:**

This study investigates the patient journey and humanistic and economic burden of axial spondyloarthritis (axSpA) in Greece, addressing a gap in real-world data on patient experiences.

**Methods:**

A cross-sectional online survey was conducted between September 2023 – February 2024, among axSpA patients 18 years old and above, members of the Greek patients’ association “Reumazin”. Data collection included socio-demographic characteristics, medical history, quality of life via Ankylosing Spondylitis Quality of Life (ASQoL) questionnaire, work productivity impairment, treatments and resource use, and patients’ expenditures over the past 12 months. Economic burden was assessed as out-of-pocket expenses and indirect cost.

**Results:**

150 patients participated (47% female; median age 57 years). The median time from symptom onset to diagnosis and to treatment initiation was 14.8 and 19 months, respectively. The most common initial symptoms were back pain (77%) and stiffness (51%). Initial specialists visited included orthopedics (53%) and pathologists/general practitioners (30%), with 63% initially misdiagnosed. The median ASQoL score was 8 (Q1: 5, Q3: 12). Among employed participants (40%), median work time impaired was 30% and activity time impaired was 40%. Mean annual out-of-pocket expenses were €400 (95% CI: €354 – €453), primarily driven by treatment costs (69%). Indirect costs were €3,321 (95% CI: €2,552 – 4,447) with presenteeism cost accounting for 84%.

**Conclusion:**

This study highlights diagnostic delays and the humanistic and economic burdens of patients living with axSpA in Greece, filling a critical gap in the literature and supporting healthcare providers and policymakers in improving patient care and optimising resource allocation.

## INTRODUCTION

Axial spondyloarthritis (axSpA) is a chronic inflammatory rheumatic disease characterised by musculoskeletal manifestations, such as back pain, peripheral arthritis, enthesitis, and dactylitis, as well as extra-musculoskeletal manifestations, including uveitis, inflammatory bowel disease, and psoriasis. The leading symptom in axSpA is inflammatory back pain.^1–3^ AxSpA exists along a spectrum that encompasses two clinical entities: radiographic axial spondyloarthritis (r-axSpA, also named ankylosing spondylitis or AS) and non-radiographic axSpA (nr-axSpA).^3^ The onset age typically ranges between 35–45 years. The global prevalence of axSpA is estimated to be between 0.3% and 1.4%. The prevalence of r-axSpA is 2 to 3 times higher in men than women, whereas nr-axSpA has a similar prevalence in both sexes.^4–6^

Several genetic, immunologic, and environmental factors are associated with axSpA aetiology. Between 75% and 89% of patients with axSpA test positive for the genetic variant major histocompatibility complex 1 human leukocyte antigen (HLA)-B27, which plays a key role in disease pathogenesis and is commonly used as a diagnostic marker in clinical practice.^7–10^ Besides genetic triggers, factors such as physical trauma, obesity, alcohol consumption, smoking, and depression have been linked to the onset of axSpA.

AxSpA has been associated with significant socioeconomic consequences. The overall humanistic burden, including the detrimental impact on patients’ physical function, work productivity, health-related quality of life (HRQoL), as well as direct and indirect costs, presents a longstanding challenge to patients, healthcare systems, and policy makers.^11^ Delayed axSpA diagnosis is linked to increased functional impairment, higher healthcare costs, and poorer quality of life (QoL), reflecting a significant clinical and socioeconomic burden as reported in various settings and current literature.^11–13^ Numerous studies have shown that diagnostic delays may last up to seven years^14–17^ In Greece, recent data from the multi-country PROOF registry reported a median diagnostic delay of 47 months (approximately four years) from the onset of chronic back pain to confirmed diagnosis, highlighting a substantial gap in timely disease recognition.^18^

While PROOF provided valuable insights into diagnostic delays, information on the broader burden of axSpA in Greece, particularly its impact on HRQoL, daily activities, and work productivity, remains scarce.^19^ Considering the economic burden, data are available only for the direct cost from a payer’s perspective. Specifically, Tsifetaki et al. conducted a cost-of-illness analysis, showing that the total annual direct cost per patient for r-axSpA reached €9,531 ± €6,322. Moreover, the authors reported that the total annual cost per patient receiving biologic agents was €10,633 ± €4,036 to €12,886 ± €920, depending on the administered agent.^20^ Although recent registry data provide important clinical insights, they do not capture the patient-reported perspective on delays or information on the associated out-of-pocket expenses and the broader socioeconomic burden.

Hence, the primary objective of our study was to depict patients journey from the onset of symptoms to diagnosis and treatment initiation. Secondary objective was to estimate the burden of axSpA among Greek patients in terms of HRQoL, productivity losses, out-of-pocket expenses, and indirect cost. By identifying key gaps and unmet needs, such as prolonged diagnostic delays and high socioeconomic burden, the findings from this study aim to enhance the understanding of these challenges and provide evidence to inform strategies for improved patient care and resource allocation.

## MATERIALS AND METHODS

### Study design

An online cross-sectional survey was conducted from September 2023 to February 2024. Eligible to participate were axSpA patients aged 18 and above, members of the Greek patients’ association “Reumazin” regardless of current treatment regimen and disease severity, provided they were fluent in Greek and had signed a consent form. Members were reached via email by the administration staff of the patients’ association. Before accessing the questionnaire, participants were presented with an online informed consent form describing the study’s objectives, voluntary nature, anonymity, and confidentiality of responses, as well as their right to withdraw at any time. Consent was obtained electronically by clicking a checkbox prior to proceeding with the questionnaire. The study protocol received approval from the management board of “Reumazin” in July 2023 and adhered to the ethical principles outlined in the Helsinki Declaration.

This study was designed and reported in accordance with the 16-item checklist for survey studies by Zimba and Gasparyan.^21^

Sample size estimation for “Time from symptom onset to rheumatologist visit” required 131 participants, based on a median time of 1.00 years (Q1–Q3: 0.5–2), with a 95% confidence level and 20% precision.^22^ Accounting for a 10% nonresponse rate, the minimum required sample size was 144 participants.

### Data collection

Data were collected via a structured questionnaire hosted on Google Forms, which was distributed to eligible patients by the “Reumazin” association (the English version is provided in Supplementary Material). Questionnaire development followed a multistep process: relevant domains were identified through literature review and consultation with clinical experts, while patient representatives contributed to refining question wording and relevance. The draft questionnaire was then pretested in a pilot study of 10 patients to assess clarity and feasibility. Based on their feedback, minor modifications were made. Face and content validity were established by an expert panel consisting of six professionals (two rheumatologists, two general practitioners, and two patient representatives), who confirmed the appropriateness and comprehensiveness of the items in capturing the study objectives.

The survey gathered information on socio-demographic characteristics (sex, age, residence, education level, current employment status, marital status, current annual income, weight, height, and smoking status), and medical history (comorbidities and family history of axSpA). The patient experiences with axSpa was assessed with a selection of 10 questions of a previously published, US web-based survey, covering symptoms, time to seek care, year of diagnosis, time to diagnosis, initial physician specialty, misdiagnosis, time to rheumatologist visit, time to first treatment, initial medications, and current physician specialty.^23^

QoL was measured using the Ankylosing Spondylitis Quality of Life (ASQoL) questionnaire for which a certified translated version of the questionnaire is available in Greek, with scores ranging from 0 to 18, where higher scores indicate poorer HRQoL.^24,25^ Work productivity impairments were assessed using the Work Productivity and Activity Impairment (WPAI) questionnaire, which provided scores for absenteeism, presenteeism, overall work productivity loss, and activity impairment outside work. Each score ranges from 0% (no impairment) to 100% (impaired all the time), with higher scores to indicate greater impairment, highlighting the condition’s impact on productivity and functioning, while lower scores to reflect a lesser impact and better management of work and daily activities.^26^ Absenteeism and presenteeism costs were derived using the corresponding items from the WPAI questionnaire. Weekly absenteeism cost was calculated by multiplying hours lost from work with mean hourly earnings (€21,297 GDP per capita in 2023 in Greece as provided by the International Monetary Fund (IMF) / 245 working days / 8 hours). Weekly presenteeism cost was estimated by multiplying affected hours with reduced productivity percentage and mean hourly earnings. Annual absenteeism and presenteeism costs were weekly costs multiplied by 49 working weeks.

Additionally, participants provided information about their current treatment, including topical therapies, non-steroidal anti-inflammatory drugs (NSAIDs), corticosteroids, conventional disease modifying anti-rheumatic drugs (DMARDs), biologic DMARDs, and targeted synthetic DMARDs, choices selected based on the current ASAS-EULAR recommendations for the management of AxSpA.^3^ Finally, patients were asked to provide details about their healthcare utilisation and out-of-pocket expenses over the previous 12 months. This encompassed data on the number and length of hospital stays, frequency of visits to private doctors or healthcare facilities, ongoing treatments, and the total out-of-pocket expenses incurred for these healthcare services related to their condition.

#### Statistical analysis

Continuous variables were summarised as means with standard deviations (SD) or as medians with interquartile ranges (1st and 3rd quartiles, Q1 – Q3). Categorical variables were described using absolute and relative frequencies. Cost data were reported as means with 95% confidence intervals (CI). Given that cost data often have a skewed distribution due to a small number of high-cost cases and are truncated at zero, the 95% CI for mean costs was estimated using 5,000 nonparametric bootstrap resamples.

For time from symptoms’ onset to treatment initiation, the proportional hazards assumption for the predictor categories was evaluated both graphically using loglog plots and by comparing the Kaplan-Meier survival curves with the predicted survival from the Cox model. Schoenfeld residuals were also assessed after fitting a Cox model. Parametric Cox models were subsequently used to identify predictors for the time from symptom onset to treatment initiation, with those showing a p-value less than 0.05 being included in a multivariate model.

Univariate and multivariate generalised linear models (GLMs) with Gaussian family and identity link function were applied to identify predictors of HRQoL. To determine factors associated with economic burden [out-of-pocket expenses, annual indirect cost (absenteeism and presenteeism costs)] GLMs with a gamma distribution and log link function were applied, which best fit the expenditure data according to the Box-Cox test and the modified Park test. Baseline demographic, lifestyle, anthropometric and clinical characteristics of patients were used as potential predictors; those with a p-value less than 0.15 were included in the multivariate models. The significance level was set to a=0.05. All analyses were conducted using STATA software (version 17.0, 2021, STATA Corp).

## RESULTS

### Patient characteristics

Out of 164 members of ‘Reumazin’ with axSpA reached, 150 participated in the survey, achieving a completion rate of 89%. Of these, 47% were female, with a median age of 57.0 years (Q1: 45.0, Q3: 65.0). Additionally, among participants with available data (n=143), 78% reported having at least one comorbidity, with arterial hypertension (36%) and hypercholesterolaemia (30%) being the most common. During the data collection period, 97% of participants were receiving treatment for axSpA; 79% were treated with b/tsDMARDs, either as monotherapy or in combination with csDMARDs, NSAIDs, corticosteroids, and/or topical treatments. Most commonly used biologic agents were the TNF-α inhibitors (73% among those under b/tsDMARDs). Conventional DMARDs either alone or in combination with NSAIDs, corticosteroids, and/or topical treatments were used by 15% of the participants (**Table 1**).

**Table 1.** Demographics, clinical profile, and treatment of study participants (N=150).

**Demographics, Clinical profile, and Treatment**	**N=150**
***Demographics – General profile***	
**Sex, n (%)**	
Female	71 (47.3%)
Male	79 (52.7%)
**Age, years**	
Median (Q1 – Q3)	57.0 (45.0 – 65.0)
**Residence, n (%)**	
Urban area	122 (81.3%)
Suburban / Rural area	28 (18.7%)
BMI* , n (%)	
Underweight / Normal range	64 (42.7%)
Overweight	71 (47.3%)
Obese	15 (10.0%)
**Marital status, n (%)**	**N=147**
Unmarried / Divorced / Widow(er)	55 (37.4%)
Married / cohabitation	92 (62.6%)
**Occupational status, n (%)**	
Freelancer / self-employed	28 (18.7%)
Employee	34 (22.7%)
Unemployed	10 (6.7%)
Retired	62 (41.3%)
Student	2 (1.3%)
Household	14 (9.3%)
**Education, n (%)**	**N=148**
Primary school / Lower secondary education / Upper secondary education	85 (57.4%)
Bachelor’s degree	49 (33.1%)
Master’s/Doctoral degree	14 (9.5%)
**Annual household income, n (%)**	**N=131**
<10,000 €	32 (24.4%)
10,000 € - 20,000 €	62 (47.3%)
20,000 € - 30,000 €	29 (22.1%)
> 30,000 €	8 (6.2%)
**Smoking habits, n (%)**	
Smoker	27 (18.0%)
Former smoker	67 (44.7%)
No smoker	56 (37.3%)
***Medical Profile***	
**Comorbidities, n (%)**	**N=143**
None	31 (21.7%)
One	31 (21.7%)
Two	31 (21.7%)
Three or more	50 (35.0%)
**Comorbidities, n (%)**	**N=143**
Arterial hypertension	52 (36.4%)
Hypercholesterolemia	43 (30.1%)
Fibromyalgia	42 (29.4%)
Osteoporosis	39 (27.3%)
Depression or anxiety disorder	26 (18.2%)
Asthma	20 (14.0%)
Coronary heart disease (heart failure, arrhythmia, heart valve problems)	16 (11.2%)
Ocular disease of autoimmune etiology (Uveitis, Keratitis, Blepharitis, Conjunctivitis, Episcleritis)	13 (9.1%)
Diabetes Mellitus	11 (7.7%)
Non-alcoholic fatty liver	6 (4.2%)
Crohn's disease or ulcerative colitis	5 (3.5%)
Cerebrovascular diseases (Stroke, Peripheral artery disease, Aortic Valve Disease)	3 (2.1%)
**Family history of axSpA,** n (%)	**N=110**
Yes	74 (67.3%)
No	36 (32.7%)
***Treatment of axSpA & Treatment perspectives***	
**Current treatment, n (%)**	**Ν=149**
Only b/tsDMARDs	54 (36.2%)
b/tsDMARDscombinations^1^	63 (42.3%)
Only csDMARDs	9 (6.0%)
csDMARDs combinations (excluding b/tsDMARDs)^2^	14 (9.4%)
Other^3^	4 (2.7%)
No treatment	5 (3.4%)
**On-going treatment among those treated, n (%)** **1**	**N=144**
Biologic agents (bDMARDs) and/or JAKs (tsDMARDs)	117 (81.3%)
Conventional DMARDs [Methotrexate, Leflunomide (Arava®), Sulfasalazine (Azulfidine®)]	62 (43.1%)
Oral corticosteroids	26 (18.1%)
Topical treatments (creams, ointments)	13 (9.0%)
NSAIDs	21 (14.6%)
Cortisone via intra-articular/local injection	11 (7.6%)
Other: Painkillers	1 (0.7%)
Agents among those under b/tsDMARDs, n (%)^**^	**N=117**
TNF-α inhibitor [Adalimumab (Humira®), Certolizumab Pegol (Cimzia®), Etanercept (Enbrel®), Golimumab (Simponi®), Infliximab (Remicade®/Remsima®/Inflectra®)]	85 (72.6%)
Interleukin-17 inhibitor [Ixekizumab (Taltz®), Secukinumab (Cosentyx®)]	21 (17.9%)
JAK kinase inhibitor [Tofacitinib (Xeljanz), Upadacitinib (Rinvoq®)]	10 (8.5%)

1 with csDMARDs, NSAIDs, cortisone, and/or topical treatments;

2 with NSAIDs, corticosteroids, and/or topical treatments;

3 NSAIDs, corticosteroids, topical treatments and/or painkillers.

* BMI was categorised as underweight (<18.5), normal range (18.5 to <25), overweight (25 to <30), and obese (≥30), according to standard World Health Organisation classifications.

** Each participant could select more than one option.

cs/b/ts DMARD: conventional/biologic/targeted synthetic disease-modifying antirheumatic drug; NSAIDs: Non-Steroidal Anti-Inflammatory Drugs; Q: quartile; TNF-α: Tumour Necrosis Factor alpha.

### Diagnosis and patient journey

Among the participants, 146 reported experiencing at least one symptom that prompted them to seek medical care. The most common symptom was back pain (77%), followed by stiffness (51%). The median age at axSpA diagnosis was 44.0 years (Q1: 36.0, Q3: 52.0). Initially, the most frequently consulted specialists were orthopaedics (53%) and pathologists/general practitioners (30%), but at the time of data collection, the vast majority (97%) were seeing a rheumatologist. Additionally, 63% of 129 participants with available data had been initially misdiagnosed. Among them, the most common diagnoses were sciatica (40%), psychosomatic (21%) and rheumatoid arthritis (15%). While 49% could not recall their first medication for axSpA, among those who did remember (n=76), the first treatments reported included conventional DMARDs (46%), corticosteroids (32%), b/tsDMARDs (24%), NSAIDs (9%), vitamins (1%) and gold salts (1%). The median (Q1 – Q3) times from the onset of the first symptom to the initial visit with a rheumatologist, to diagnosis, and to treatment initiation were 12.5 (7.0 – 24.5) months, 14.8 (8.0 – 29.5) months, and 19.0 (10.3 – 31.5) months, respectively (**Table 2**).

**Table 2.** Patients’ journey from symptoms onset to treatment initiation.

**Pathway to axSpA diagnosis**	**N=150**
**Symptoms that led you to seek medical care, n (%)**	**N=146**
Back Pain	112 (76.7%)
Stiffness	75 (51.4%)
Low Back Pain	68 (46.6%)
Fatigue	60 (41.1%)
Neck Pain	59 (40.4%)
Joint Pain	58 (39.7%)
Sleep Problems	52 (35.6%)
Difficulty Walking	40 (27.4%)
Tendon/Ligament Pain	28 (19.2%)
Chest Pain	25 (17.1%)
Swollen Joints	24 (16.4%)
Eye Problems	24 (16.4%)
Pelvic Pain	10 (6.8%)
Shortness of Breath	9 (6.2%)
Skin Rash/Psoriasis	5 (3.4%)
Foot Problems (e.g., sole, toes, etc.)	0 (0.0%)
Psychological Issues (Anxiety/Depression)	0 (0.0%)
**Specialty of the physician you first visited, n (%)**	**N=148**
Orthopaedist	78 (52.7%)
Pathologist/General Practitioner	44 (29.7%)
Rheumatologist	25 (16.9%)
Ophthalmologist	1 (0.7%)
Dermatologist	0 (0.0%)
**Initial misdiagnosis, n (%)** **1**	**N=129**
Never received an incorrect diagnosis	48 (37.2%)
Sciatica	39 (30.2%)
Psychosomatic	20 (15.5%)
Rheumatoid Arthritis	15 (11.6%)
Osteoarthritis	10 (7.8%)
Other	7 (5.4%)
Fibromyalgia	5 (3.9%)
Gout	1 (0.8%)
**What specialty has the physician currently seeing you for axSpA, n (%)**	**N=149**
Rheumatologist	145 (97.3%)
Orthopaedist	3 (2.0%)
Pathologist/General Practitioner	1 (0.7%)
Dermatologist	0 (0.0%)
**Time Intervals, months**	
Time from Symptom Onset to 1^st^ medical seek	**N=147**
Median (Q1 – Q3)	5.0 (2.0 – 9.5)
Time from Symptom Onset to Rheumatologist Visit	
Median (Q1 – Q3)	12.5 (7.0 – 24.5)
Time from Symptom Onset to Diagnosis	**N=144**
Median (Q1 – Q3)	14.8 (8.0 – 29.5)
Time from Symptom Onset to Treatment Initiation	**N=144**
Median (Q1 – Q3)	19.0 (10.3 – 31.5)
Time from 1^st^ medical seek to Rheumatologist Visit	
Median (Q1 – Q3)	5.0 (2.0 – 12.0)
Time from 1^st^ medical seek to Diagnosis	**N=147**
Median (Q1 – Q3)	6.0 (3.0 – 12.0)
Time from Diagnosis to Treatment Initiation	
Median (Q1 – Q3)	1.5 (1.0 – 3.0)

1 excluding the answer “Never received an incorrect diagnosis”, a patient could have reported more than one initial diagnosis. axSpA: axial spondyloarthritis; Q: quartile.

In the univariate models, several factors were associated with increased time from symptoms’ onset to treatment initiation, including residing in rural or suburban areas instead of urban areas, having arterial hypertension, autoimmune-related ocular disease, fibromyalgia, joint pain, swollen joints, eye problems, sleep problems as initial symptoms, and visiting a pathologist/general practitioner instead of a rheumatologist. Conversely, older age and having a bachelor’s or a master’s/doctoral degree versus primary, or secondary education were associated with shorter times to treatment initiation. In the multivariate model, older age, suburban/rural areas instead of urban ones, having arterial hypertension, and experiencing joint pain as an initial symptom remained significant predictors of the time to treatment initiation (**Table 3**).

**Table 3.** Factors associated with time from symptoms onset to treatment initiation.

**Time from onset of the first symptom until treatment initiation, in months**	**Univariate models**	**Multivariate model**				

		**N=135**				

	**HR (95% CI)**	**p**	**HR (95% CI)**	**p**
**Age at diagnosis, in years**	**N=143**			
	1.02 (1.001, 1.03)	**0.041**	1.02 (1.002, 1.04)	**0.033**
**Sex**	**N=144**			
Female vs. Male	1.32 (0.93, 1.87)	0.116		
**BMI**	**N=144**			
Overweight vs. Underweight/Normal range	1.19 (0.83, 1.71)	0.171		
Obese vs. Underweight/Normal range	0.67 (0.36, 1.25)			
**Residence, n (%)**	**N=144**			
Suburban/rural area vs. Urban area	0.53 (0.34, 0.82)	**0.005**	0.45 (0.26, 0.80)	**0.007**
**Education, n (%)**	**N=143**			
Bachelor’s degree vs. Primary school/Lower-Upper secondary education	1.54 (1.06, 2.23)	**0.008**	1.62 (1.002, 2.65)	0.061
Master’s/Doctoral degree vs. Primary school/Lower-Upper secondary education	2.17 (1.21, 3.91)		1.99 (0.95, 4.20)	
**Smoking habits, n (%)**	**N=144**			
Former smoker vs. Smoker	0.76 (0.48, 1.21)	0.162		
No smoker vs. Smoker	1.08 (0.67, 1.74)			
**Comorbidities (Each vs. no)**	**N=139**			
Coronary heart disease	0.67 (0.40, 1.15)	0.146		
Arterial hypertension	0.47 (0.32, 0.69)	**< 0.001**	0.52 (0.33, 0.82)	**0.005**
Cerebrovascular diseases	1.55 (0.37, 6.53)	0.551		
Diabetes Mellitus	1.18 (0.61, 2.28)	0.629		
Asthma	1.17 (0.71, 1.93)	0.532		
Hypercholesterolemia	0.71 (0.49, 1.04)	0.078		
Osteoporosis	0.81 (0.55, 1.19)	0.280		
Crohn's disease or ulcerative colitis	1.41 (0.56, 3.54)	0.464		
Ocular disease of autoimmune etiology	0.43 (0.22, 0.81)	**0.009**	0.91 (0.43, 1.87)	0.777
Non-alcoholic fatty liver	1.82 (0.78, 4.28)	0.167		
Fibromyalgia	0.54 (0.37, 0.80)	**0.002**	1.08 (0.67, 1.74)	0.757
Depression or anxiety disorder	1.04 (0.67, 1.62)	0.851		
**Symptoms that led to seek medical care (Each vs. no)**	**N=142**			
Back Pain	0.92 (0.61, 1.37)	0.668		
Low Back Pain	0.85 (0.61, 1.19)	0.346		
Foot Problems (e.g., sole, toes, etc.)				
Joint Pain	0.66 (0.46, 0.93)	**0.017**	0.60 (0.39, 0.92)	**0.019**
Swollen Joints	0.57 (0.36, 0.91)	**0.018**	0.68 (0.37, 1.25)	0.215
Neck Pain	0.86 (0.61, 1.21)	0.385		
Difficulty Walking	0.93 (0.63, 1.36)	0.702		
Stiffness	0.80 (0.56, 1.14)	0.223		
Eye Problems	0.44 (0.27, 0.72)	**0.001**	1.04 (0.56, 1.93)	0.898
Fatigue	0.86 (0.61, 1.20)	0.371		
Skin Rash/Psoriasis	0.55 (0.20, 1.53)	0.253		
Tendon/Ligament Pain	0.67 (0.43, 1.04)	0.075		
Pelvic Pain	1.40 (0.72, 2.71)	0.319		
Chest Pain	1.10 (0.70, 1.72)	0.690		
Psychological Issues (Anxiety/Depression)				
Sleep Problems	0.58 (0.41, 0.84)	**0.003**	0.78 (0.51, 1.18)	0.241
Shortness of Breath	0.80 (0.39, 1.66)	0.556		
Other^1^				
**Specialty of the physician you first visited**	**N=143**			
Pathologist/General Practitioner vs. Rheumatologist	0.46 (0.27, 0.77)	**0.013**	0.49 (0.25, 0.94)	0.094
Orthopedic vs. Rheumatologist	0.64 (0.40, 1.03)		0.71 (0.40, 1.28)	
**Have you ever been misdiagnosed with a condition other than axSpA**	**N=127**			
Yes vs. No	0.70 (0.48, 1.02)	0.060		

Cox regression for time-to-treatment initiation; Variables with p<0.05 were entered in the multivariate model. P-values represent the overall significance of each variable in the model (Wald test). Bolded p-values represent statistically significant associations.

axSpA: axial spondyloarthritis; CI: confidence interval; HR: hazard ratio; p: p-value.

#### Quality of life and work productivity

**Figure 1.**
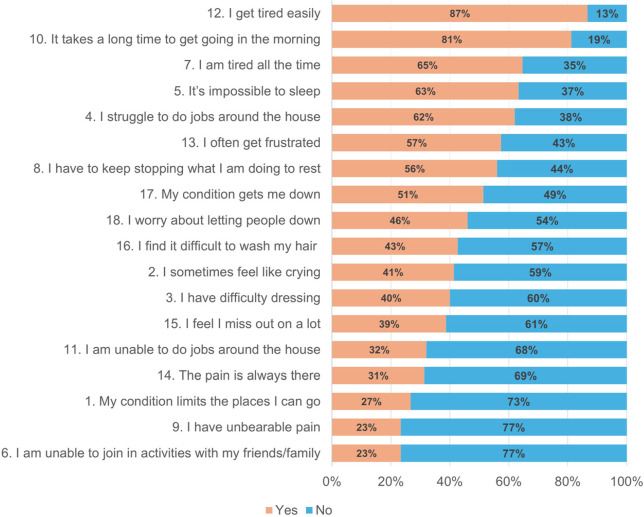
Responses to individual Ankylosing Spondylitis Quality of Life (ASQoL) items (N=148). Alt text: A horizontal bar chart showing the percentage of respondents who answered 'Yes' (orange) or 'No' (blue) to various ASQoL statements. The most frequently endorsed statements include 'I get tired easily' (87% Yes), 'It takes a long time to get going in the morning' (81% Yes), and 'I am tired all the time' (65% Yes), while the least endorsed statements include 'I have unbearable pain' (23% Yes) and 'I am unable to join in activities with my friends/family' (23% Yes). The chart illustrates the impact of Ankylosing Spondylitis on fatigue, physical functioning, and emotional well-being among respondents.

The participants’ responses to the ASQoL questionnaire are presented in **Figure 1**. The median ASQoL score was 8.0 (Q1: 5.0, Q3: 12.0) [mean (SD)=8.7 (4.7)]. In the univariate analysis, older age, being female, obese, having osteoporosis or fibromyalgia, and depression or anxiety disorder were associated with lower quality of life. Current treatment was also associated with HRQoL; specifically, those on a combination treatment of biologics with other agents (DMARDs and/or corticosteroids and/or topical treatments and/or NSAIDs) had more impaired HRQoL compared to those under only biologic agents, and those under only DMARDs had better HRQoL compared to those under only biologic agents. In the multivariate model, significant factors included BMI, osteoporosis and current treatment (**Table 4**).

**Table 4.** Factors associated with patients’ quality of life.

	**Univariate models**	**Multivariate model**				
		
			**N=142**	

**ASQoL**	**Coef. (95% CI)**	**p**	**Coef. (95% CI)**	**p**
**Age, in years**	**N=150**			
1-yr increase	0.08 (0.02, 0.14)	**0.005**	0.05 (−0.01, 0.11)	0.101
**Age at diagnosis, in years**	**N=150**			
1-yr increase	0.05 (−0.02, 0.11)	0.164		
**Time from onset of the first symptom to diagnosis**	**N=144**			
1-yr increase	0.01 (−0.01, 0.03)	0.371		
**Sex**	**N=150**			
Female vs. Male	2.41 (0.95, 3.87)	**0.001**	0.93 (−0.68, 2.53)	0.258
**BMI**	**N=150**			
Overweight vs. Underweight/Normal range	−0.70 (−2.24, 0.84)	**0.004**	−0.51 (−1.96, 0.95)	**0.035**
Obese vs. Underweight/Normal range	3.63 (1.06, 6.19)		2.80 (0.36, 5.24)	
**Smoking habits**	**N=150**			
Former smoker vs. Smoker	1.49 (−0.61, 3.59)	0.373		
No smoker vs. Smoker	1.21 (−0.94, 3.37)			
**Comorbidities (Yes vs. No)**	**N=143**			
Coronary heart disease	−0.08 (−2.56, 2.39)	0.947		
Arterial hypertension	1.09 (−0.52, 2.70)	0.186		
Cerebrovascular diseases	−0.65 (−6.09, 4.79)	0.815		
Diabetes Mellitus	2.27 (−0.64, 5.17)	0.126		
Asthma	0.95 (−1.30, 3.19)	0.408		
Hypercholesterolemia	−0.18 (−1.88, 1.52)	0.837		
Osteoporosis	3.81 (2.18, 5.45)	**< 0.001**	1.87 (0.05, 3.69)	**0.044**
Crohn's disease or ulcerative colitis	−0.87 (−5.11, 3.37)	0.689		
Ocular disease of autoimmune etiology	0.40 (−2.31, 3.11)	0.772		
Non-alcoholic fatty liver	−1.36 (−5.24, 2.52)	0.492		
Fibromyalgia	2.03 (0.35, 3.71)	**0.018**	0.28 (−1.33, 1.90)	0.729
Depression or anxiety disorder	4.30 (2.41, 6.19)	**< 0.001**	1.98 (−0.03, 3.99)	0.054
**Current treatment**	**N=149**			
b/tsDMARDs combinations^1^ vs. Only b/tsDMARDs				
	2.70 (1.06, 4.33)	**0.001**	2.42 (0.86, 3.98)	**< 0.001**
Only csDMARDs vs. Only b/tsDMARDs	−4.00 (−7.09, −0.91)		−3.47 (−6.29, −0.65)	
csDMARDs combinations (excluding b/tsDMARDs)^2^ vs. Only b/tsDMARDs	1.90 (−0.67, 4.48)		−1.15 (−1.31, 3.62)	
Other^3^ vs. Only b/tsDMARDs	−1.42 (−5.86, 3.03)		−2.82 (−7.44, 1.80)	
No treatment vs. Only b/tsDMARDs	0.33 (−3.68, 4.34)		−0.96 (−5.17, 3.25)	

1 with csDMARDs, NSAIDs, corticosteroids, and/or topical treatments;

2 with NSAIDs, corticosteroids, and/or topical treatments;

3 NSAIDs, corticosteroids, topical treatments and/or painkillers.

GLM with gaussian family and identity link function with dependent variable the ASQoL score (higher scores indicate poorer quality of life). Variables with p<0.15 were entered in the multivariate model. P-values represent the overall significance of each variable in the model (Wald test). Bolded p-values represent statistically significant associations.

ASQoL: Ankylosing Spondylitis Quality of Life; CI: confidence interval; Coef: coefficient; cs/b/ts DMARD: conventional/biologic/targeted synthetic disease-modifying antirheumatic drug; GLM: generalised linear model; NSAIDs: Non-Steroidal Anti-Inflammatory Drugs; p: p-value.

Of the participants, 40% were employed at the time of the survey. Among these employed participants, the median percentage of work time missed due to ax-SpA (absenteeism) was 0% (Q1: 0%, Q3: 5%), while the median percentage of impairment due to axSpA while working (presenteeism) was 30% (Q1: 20%, Q3: 40%). The median overall work impairment due to axSpA was 30% (Q1: 20%, Q3: 50%) of their total work time, and the median activity impairment was 40% (Q1: 30%, Q3: 60%) (data not shown).

### Healthcare utilisation and economic burden

In the past 12 months, 63% of participants visited a private doctor, and 6% were hospitalised, with a median hospitalisation duration of 5.0 (Q1: 4.0, Q3: 10.0) days (**Table 5**).

**Table 5.** Outpatient visits and hospitalisations during the last 12 months (N=150).

**Outpatient visits & hospitalisations last 12 months**	**N=150**
**Outpatient visits, n (%)**	
Private doctor	85 (56.7%)
Private doctor (Public sector)	10 (6.7%)
Hospital appointment (morning)	66 (44.0%)
Hospital appointment (evening)	29 (19.3%)
**Median (Q1 – Q3) number of outpatient visits/patient**	
Private doctor (N=102)	3.0 (2.0 – 5.0)
Private doctor (Public sector) (N=10)	3.5 (2.0 – 9.0)
Hospital appointment (morning) (N=44)	5.0 (3.0 – 8.0)
Hospital appointment (evening) (N=37)	2.0 (2.0 – 4.0)
**Hospitalised, n (%)**	
Yes	9 (6.0%)
No	141 (94.0%)
**Median (Q1 – Q3) number of hospitalisations/patient**	**N=9**
Public sector	1.0 (1.0 – 1.0)
Private sector	0.0 (0.0 – 0.0)
**Median (Q1 – Q3) duration of hospitalisations, days**	5.0 (4.0 – 10.0)

Q: quartile.

The mean out-of-pocket expenses per patient was estimated at €400 (95% CI: €354 – €453). Treatment costs were the main driver (69%), averaging €275 (95% CI: €239 – €323), followed by outpatient visits at €125 (95% CI: €103 – €152). Visits to private doctors constituted 70% of outpatient visit costs, averaging €87 (95% CI: €71 – €107) (**Table 6**). Univariate analysis showed that having osteoporosis or depression or anxiety disorder was associated with higher direct healthcare costs, while being overweight vs. underweight/normal range or having asthma was associated with lower direct healthcare costs. Being overweight or having osteoporosis remained significant in the multivariate analysis (**Table 7**).

**Table 6.** Annual out-of-pocket expenses and indirect cost per patient of axSpA (N=141).

**Annual cost, *Mean (95% CI)***	**Cost, Mean (95% CI)**
**Total out-of-pocket expenses/patient, €**	**400 (354 – 453)**
**Cost of outpatient visits/patient, €**	125 (103 – 152)
Private doctor	87 (71 – 107)
Private doctor (via EOPYY)	8 (3 – 18)
Hospital appointment (morning)	0 (0 – 0)
Hospital appointment (evening)	30 (20 – 43)
**Cost of hospitalisations/patient, €**	0 (0 – 0)
**Cost of any treatment/patient, €**	275 (239 – 323)
**Indirect cost/patient, €**	**3,322 (2,552 – 4,447)**
Absenteeism cost, €	544 (298 – 1,133)
Presenteeism cost, €	2,778 (2,163 – 3,551)

CI: confidence interval; EOPPY: Greek National Organisation for Healthcare Services Provision.

**Table 7.** Factors associated with annual out-of-pocket expenses and indirect cost per patient (Univariate and multiple models).

	**Out-of-pocket expenses**				**Indirect total cost**								

	**Univariate models**		**Multivariate model**		**Univariate models**		**Multivariate model**	

			**N=135**				**N=135**	

**Annual cost the last 12 month**s	**Coef. (95% CI)**	**p**	**Coef. (95% CI)**	**p**	**Coef. (95% CI)**	**p**	**Coef. (95% CI)**	**p**
**Age, in years**	**N=141**				**N=141**			
1-yr increase	−0.003 (−0.01, 0.01)	0.505			−0.15 (−0.25, −0.06)	**0.002**	−0.25 (−0.42, −0.08)	**0.004**
**Time from onset of the first symptom to diagnosis**	**Ν=135**				**N=135**			
1-yr increase	−0.001 (−0.004, 0.003)	0.763			−0.03 (−0.05, −0.01)	**0.001**	−0.01 (−0.05, 0.03)	0.618
**Sex**	**N=141**				**N=141**			
Female vs. Male	0.10 (−0.15, 0.35)	0.427			−0.11 (−0.65, 0.43)	0.680		
**BMI**	**N=141**				**N=141**			
Overweight vs. Underweight/Normal range	−0.50 (−0.75, −0.26)	**<0.001**	−0.44 (−0.73, −0.16)	**0.008**	0.02 (−0.56, 0.60)	0.984		
Obese vs. Underweight/Normal range	−0.21 (−0.61, 0.19)		−0.27 (−0.71, 0.17)		0.09 (−0.85, 1.02)			
**Residence**	**N=141**				**N=141**			
Suburban/rural area vs. Urban area	−0.23 (−0.54, 0.08)	0.142	−0.23 (−0.56, 0.10)	0.164	−0.56 (−1.25, 0.13)			
**Education**	**N=139**				**N=139**			
Bachelor’s degree vs. Primary school/Lower-Upper secondary education	−0.01 (−0.29, 0.27)	0.911			0.36 (−0.30, 1.01)	0.194		
Master’s/Doctoral degree vs. Primary school/Lower-Upper secondary education	0.09 (−0.34, 0.52)				0.86 (−0.15, 1.87)			
**Smoking habits**	**N=141**				**N=141**			
Former smoker vs. Smoker	0.43 (0.08, 0.78)	0.054	0.13 (−0.23, 0.49)	0.755	−0.29 (−1.05, 0.46)	0.486		
No smoker vs. Smoker	0.35 (−0.01, 0.70)		0.12 (−0.24, 0.47)		−0.47 (−1.24, 0.30)			
**Comorbidities (Each vs. No)**	**N=135**				**N=135**			
Coronary heart disease	0.18 (−0.23, 0.58)	0.390			−1.67 (−2.61, −0.72)	**0.001**	−2.55 (−4.56, −0.54)	**0.013**
Arterial hypertension	0.06 (−0.21, 0.34)	0.640			−0.09 (−0.70, 0.51)	0.763		
Cerebrovascular diseases	−0.16 (−1.04, 0.72)	0.720			−0.15 (−2.08, 1.78)	0.879		
Diabetes Mellitus	−0.01 (−0.56, 0.54)	0.963			−0.65 (−1.86, 0.56)	0.291		
Asthma	−0.49 (−0.875, −0.10)	**0.013**	−0.33 (−0.71, 0.04)	0.081	−0.76 (−1.63, 0.12)	0.089	−1.61 (−3.87, 0.65)	0.162
Hypercholesterolemia	0.23 (−0.06, 0.52)	0.124	0.26 (−0.02, 0.55)	0.071	−0.62 (−1.23, −0.01)	**0.047**	−0.49 (−2.08, 1.11)	0.550
Osteoporosis	0.42 (0.14, 0.70)	**0.003**	0.33 (0.02, 0.63)	**0.034**	−0.58 (−1.23, 0.06)	0.075	−1.74 (−3.17, −0.32)	**0.017**
Crohn's disease or ulcerative colitis	0.30 (−0.47, 1.07)	0.445			0.05 (−1.63, 1.74)	0.951		
Ocular disease of autoimmune aetiology	0.24 (−0.23, 0.70)	0.317			−0.07 (−1.07, 0.92)	0.883		
Non-alcoholic fatty liver	−0.26 (−0.88, 0.36)	0.414			−1.57 (−2.92, −0.22)	**0.023**	−5.45 (−8.75, −2.16)	**0.001**
Fibromyalgia	0.26 (−0.02, 0.54)	0.071	0.04 (−0.25, 0.34)	0.783	−0.02 (−0.65, 0.60)	0.940		
Depression or anxiety disorder	0.37 (0.06, 0.69)	**0.022**	0.11 (−0.22, 0.44)	0.496	−0.08 (−0.82, 0.65)	0.821		
**Current Treatment**					**N=140**			
b/tsDMARDs combinations^1^ vs. Only b/tsDMARDs	0.12 (−0.16, 0.40)	0.350			0.06 (−0.55, 0.66)	0.800		
Only csDMARDs vs. Only b/tsDMARDs	−0.27 (−0.82, 0.28)				−0.15 (−1.36, 1.05)			
csDMARDs combinations (excluding b/tsDMARDs)^2^ vs. Only b/tsDMARDs	0.20 (−0.24, 0.64)				0.02 (−0.94, 0.98)			
Other^3^ vs. Only b/tsDMARDs	0.68 (−0.08, 1.44)				0.49 (−1.16, 2.14)			
No treatment vs. Only b/tsDMARDs	0.16 (−0.59, 0.92)				1.16 (−0.49, 2.81)			

1 with cDMARDs, NSAIDs, corticosteroids, and/or topical treatments;

2 with NSAIDs, corticosteroids, and/or topical treatments;

3 NSAIDs, corticosteroids, topical treatments and/or painkillers.

GLM with gamma family and a log link function. All variables with p < 0.15 in the univariate model, were inserted to the multivariate model. P-values represent the overall significance of each variable in the model. (Wald test). Bolded p-values represent statistically significant associations.

CI: confidence interval; Coef: coefficient; cs/b/ts DMARD: conventional/biologic/targeted synthetic disease-modifying antirheumatic drug; GLM: generalised linear model; NSAIDs: Non-Steroidal Anti-Inflammatory Drugs; p: p-value.

Indirect costs, mainly from presenteeism (84%), averaged €3,321 (95% CI: €2,552 – €4,447) (**Table 6**). Univariate analysis indicated that increasing age, prolonged time from symptom onset to diagnosis, and the presence of coronary heart disease, hypercholesterolemia, and non-alcoholic fatty liver were associated with lower total annual costs. Multivariate analysis confirmed that age, coronary heart disease and non-alcoholic fatty liver significantly impact the indirect costs. Having osteoporosis was as well associated with lower indirect cost in the multivariate model (**Table 7**).

## DISCUSSION

To our knowledge, this is the first extensive effort to assess the diagnostic journey of patients with axSpA in Greece, including delays from symptom onset to diagnosis and treatment, as well as its impact on HRQoL, work productivity, and out-of-pocket expenditures. Our findings on the diagnostic journey of patients with ax-SpA reveal significant delays from symptom onset to treatment initiation. The median time from symptom onset to first medical consultation was 5.0 months. Notably, the median time from symptom onset to diagnosis was 14.8 months, and the median time from diagnosis to first treatment was 1.5 months.

Comparatively, the literature reports varying diagnostic delays. Results from the Spondyloarthritis Italian Registry indicated that the median time from symptom onset to diagnosis was 36 months for axial SpA and 24 months for peripheral SpA patients.^27^ This substantial difference in our cohort, with a median time of 14.8 months, may be attributed to differences in healthcare systems, patient awareness, and diagnostic practices across regions. Additionally, data from the Swiss Clinical Quality Management (SCQM) registry showed a mean diagnostic delay of 5.6 years for axSpA without psoriasis and 6.2 years for axSpA with psoriasis, indicating longer delays compared to our findings.^28^ The systematic review of Hay et al. (2022) provides a range of diagnostic delays across different countries. For instance, in France, the median diagnostic delay for axSpA was reported to be 2 years, while in South Korea, it was as long as 8 years. The median diagnostic delay in Europe was reported to be around 4 years.^17^ Furthermore, the European Map of Axial Spondyloarthritis (EMAS) study, using a cross-sectional survey, reported a mean diagnostic delay of 7.4 years, although the mean is not the most preferred measure of central tendency for axSpA.^15,29^ More recently, results from the PROOF registry reported a median diagnostic delay of 47 months (approximately 4 years) in the Greek cohort, which is longer than the delay observed in our patient-reported data.^18^ Differences between registry-based and survey-based estimates may reflect variations in methodology, recall bias, or patient selection, but both highlight the persistent challenge of delayed diagnosis in Greece. These comparisons emphasise the variability in diagnostic timelines across different regions and healthcare settings, underscoring the need for improved awareness, early recognition, and timely referral to specialists to reduce delays and enhance patient outcomes in axSpA management.

Our findings on the HRQoL among patients with axSpA indicate a notable impact of the disease. The mean (SD) and median (Q1 – Q3) ASQoL scores in our cohort were 8.7 (4.7) and 8.0 (5.0 – 12.0), respectively. Literature data from a cross-sectional study, conducted in hospital patients in Madrid, including ankylosing spondylitis and undifferentiated SpA, reported ASQoL scores with a mean of 6.0 (SD: 5.2) in a cohort of 106 patients.^30^ While these scores are slightly lower than those observed in our study, they provide a useful comparison for understanding the burden of disease on HRQoL in SpA patients.

A systematic literature review (SLR) identified studies that reported work productivity using the WPAI questionnaire in patients with axSpA initiating biologic or targeted synthetic disease-modifying antirheumatic drugs (b/tsDMARDs). The review found that approximately 57.6% to 91.4% of patients with radiographic ax-SpA (r-axSpA) and 60.0% to 74.0% with nr-axSpA were employed on a full-time or part-time basis. For working patients initiating b/tsDMARDs, the mean baseline overall work productivity impairment was 52%, absenteeism was 11% and presenteeism was 49%, while activity impairment was 58%.^27^ In comparison, we found a lower employment rate (47%) compared to the range reported in the review. This might reflect differences in the study populations or healthcare systems. Additionally, our study reports significantly lower median overall work productivity impairment (30%), absenteeism (0%), presenteeism (30%), and activity impairment (40%) rates compared to the corresponding means reported in the review. These differences might be attributed to variations in study populations, healthcare systems, treatment approaches, and data collection methodologies. However, the overall trends indicate that while our cohort experiences significant work productivity and activity impairment due to axSpA, the extent of these impairments appears to be less severe compared to the findings reported in the systematic literature review.

In a cross-sectional study conducted in Spain the mean annual indirect cost due to work productivity losses was estimated at €3,851, a finding comparable to our estimate of €3,321.^13^ In contrast, Rudwaleit et al. (2023) in a SLR, reported a much higher annual indirect cost of €31,646.09 for patients with axSpA before initiating biologic or targeted synthetic DMARDs, with a significant drop to €13,128.21 after 12–16 weeks of treatment with these advanced therapies.^27^ Several factors contribute to the substantial difference in indirect cost estimates between our study and the review by Rudwaleit et al. Our study involved long-term members of a patient organisation, likely reflecting more stable treatment regimens and fewer acute episodes, lowering costs. Additionally, our cost estimates used lower hourly wages (€10.9 vs. €29.1) and fewer working weeks (49 vs. 52.1), reflecting different economic assumptions. The higher assumed earnings and more extensive working weeks in the review naturally lead to higher estimated indirect costs, reflecting different economic contexts and possibly varying workforce productivity metrics. Overall, our findings suggest that while the indirect costs of axSpA can be substantial, they are influenced by various factors, including disease duration, treatment strategies, and economic assumptions. In the multivariate analysis, overweight status and osteoporosis were significantly associated with out-of-pocket expenses. A possible explanation is that overweight patients may be more frequently in contact with healthcare providers due to comorbidities, which could facilitate earlier recognition of axSpA symptoms and reduce the need for additional privately funded consultations or diagnostic procedures. In contrast, patients with osteoporosis are likely to incur higher out-of-pocket expenses due to more frequent specialist visits, additional investigations, and long-term treatments for bone health management that may not be fully reimbursed. Regarding indirect costs, older patients and those with cardiometabolic comorbidities (coronary heart disease and non-alcoholic fatty liver disease) were associated with lower productivity-related expenditures, likely reflecting reduced employment participation or retirement in these subgroups. Similarly, patients with osteoporosis showed lower indirect costs, which may also be explained by limited labour force participation. These associations should be interpreted with caution, as residual confounding and the relatively small sample size may also have influenced the results.

This study’s primary strength is its comprehensive examination of the experiences of patients with axSpA, encompassing aspects such as comorbidities and economic burden, thereby offering valuable insights into the diverse challenges these patients face. The adequate sample size and detailed questionnaire facilitated a thorough understanding of the disease’s impact on quality of life, work productivity, and healthcare utilisation. However, several limitations must be acknowledged. The cross-sectional design restricts the ability to establish causal relationships between variables. The use of an online survey could have introduced selection bias, excluding individuals without internet access or those less proficient with technology, potentially skewing the results. Recall bias is also a concern since the data depend on self-reported information from participants, particularly regarding medical history and treatment timelines. Additionally, the study’s focus on members of a single patient association may limit the generalisability of the findings to the broader axSpA population. Although the completion rate was high (89%), non-respondents were not analysed, which may further limit generalisability. Another limitation is the absence of clinical data, such as disease activity indices (e.g., BASDAI, ASDAS) and differentiation between radiographic and non-radiographic axSpA, since the survey relied on self-reports rather than clinical examination or imaging. In Greece, patients can directly access specialists without a formal referral pathway. However, this self-referral model may result in initial consultations with non-rheumatology specialists (e.g., orthopedists, dermatologists), delaying correct diagnosis. Furthermore, the uneven distribution of rheumatologists, concentrated mainly in urban areas, together with the limited availability of appointments in the public sector and long waiting lists, may further contribute to diagnostic delays, particularly for patients in rural regions. Seasonal variations in disease symptoms and healthcare utilisation over the eight-month data collection period could have influenced the results. Lastly, the economic burden estimates might be underestimated due to the incomplete capture of treatment cost data from the payer perspective, as only out-of-pocket expenses were considered.

This study provides important and current insights into the diagnostic delays, as well as the humanistic and economic burdens experienced by patients with axSpA in Greece, filling a significant gap in the current literature. The results can aid healthcare providers and policymakers in making more informed decisions aimed at enhancing patient care and optimising resource allocation.

## Data Availability

The datasets generated and/or analysed during the current study are not publicly available but are available from the corresponding author upon reasonable request.
